# An insight to potential application of synbiotic edible films and coatings in food products

**DOI:** 10.3389/fnut.2022.875368

**Published:** 2022-07-27

**Authors:** Sahar Seyedzade Hashemi, Nasim Khorshidian, Mehrdad Mohammadi

**Affiliations:** ^1^Department of Food Science and Technology, Faculty of Nutrition Sciences and Food Technology, National Nutrition and Food Technology Research Institute, Shahid Beheshti University of Medical Sciences, Tehran, Iran; ^2^Department of Food Technology Research, Faculty of Nutrition Sciences and Food Technology, National Nutrition and Food Technology Research Institute, Shahid Beheshti University of Medical Sciences, Tehran, Iran

**Keywords:** edible packaging, probiotic, prebiotic, synbiotic, survival

## Abstract

Edible films and coatings have gained significant consideration in recent years due to their low cost and decreasing environmental pollution. Several bioactive compounds can be incorporated into films and coatings, including antioxidants, antimicrobials, flavoring agents, colors, probiotics and prebiotics. The addition of probiotics to edible films and coatings is an alternative approach for direct application in food matrices that enhances their stability and functional properties. Also, it has been noted that the influence of probiotics on the film properties was dependent on the composition, biopolymer structure, and intermolecular interactions. Recently, the incorporation of probiotics along with prebiotic compounds such as inulin, starch, fructooligosaccharide, polydextrose and wheat dextrin has emerged as new bioactive packaging. The simultaneous application of probiotics and prebiotics improved the viability of probiotic strains and elevated their colonization in the intestinal tract and provided health benefits to humans. Moreover, prebiotics created a uniform and compact structure by filling the spaces within the polymer matrix and increased opacity of edible films. The effects of prebiotics on mechanical and barrier properties of edible films was dependent on the nature of prebiotic compounds. This review aims to discuss the concept of edible films and coatings, synbiotic, recent research on synbiotic edible films and coatings as well as their application in food products.

## Introduction

Nowadays, increasing public awareness about the relationship between diet and health resulted in producing various food products containing bioactive ingredients such as antioxidants, antimicrobials, essential macro and micronutrients, prebiotics, and probiotics (1, 2). Utilization of prebiotics and probiotics individually or in combination is increasing due to the consumers' demand for healthful products, consequently leading to the development of functional food products (3).

The Food and Agriculture Organization (FAO) and World Health Organization (WHO) defined probiotics as live microorganisms that confer health benefits to the host when taken in appropriate amounts (10^6^ to 10^7^ CFU/g) (4). Probiotics are extensively incorporated into functional food products such as dairy, cereal, meat, fruits and vegetable-based products, which exhibit health benefits and techno-functional properties (5, 6). It has been revealed that the presence of non-digestible carbohydrates or prebiotics can improve the stability and viability of probiotics in food products and the gastrointestinal tract in addition to their beneficial effects on human health (7). The combination of probiotics and prebiotics is known as synbiotic, in which the prebiotic component enhances the probiotics' growth and survival (8).

Adverse conditions during food processing (mechanical, heat, acidic and osmotic stress) and storage (water vapor transmission and oxygen) lead to insufficient delivery of viable probiotic cells, which undergo low pH and bile in the gastrointestinal tract (9). One of the recent procedures to maintain microorganisms' level at the recommended dose is embedding living cells in a low humidity bed. A unique approach can be inserting probiotics in a plasticized thin layer of a natural polymer called edible film (9–15).

Biopolymer packaging is an eco-friendly system that prevents food deterioration and enhances its quality by protecting against gases and moisture. They can carry bioactive compounds like vitamins, enzymes, antioxidants and eventually release them into the food product (16–18). In the case of probiotic edible coatings, the release is not required since the coating is assumed to be eaten with the food (19). Also, due to the antimicrobial capacity of probiotic bacteria, they may be employed as an alternative strategy to control pathogenic microorganisms (14, 20–23). In order to enhance probiotics viability, prebiotic compounds have been incorporated into film-forming solutions. It has been declared that prebiotics remarkably boosted the probiotic viability during storage and in simulated gastrointestinal conditions (8, 14, 24–26). It has also been reported that synbiotic edible films and coatings positively influenced the microbial and physicochemical quality of the food product. The studies regarding synbiotic edible packaging and its application in food products are limited and should be more explored. The present review highlights the nature of probiotics and prebiotics, their incorporation into films and coatings, characterization of developed packaging, viability of probiotics in synbioitic films and coatings as well as their application in food products.

## Probiotics, prebiotics and synbiotics

The word probiotic is a derivative of the Greek word “probius” meaning life-giving. It is conceptually opposite to “antibiotic,” meaning anti-life (27). During the 1960s, an increasing attention was paid to the supplements containing live bacteria to reduce the widespread use of antibiotics and their side effects on farm animals. Lilly and Stillwell, in 1965, first described the word probiotic as secretions from protozoan that stimulated the growth of another (28). Probiotics are live microorganisms which exhibit health advantages to the host at a specific concentration. Most of the microorganisms currently used as probiotics belong to species of the genera *Lactobacillus* and *Bifidobacterium*, but several other genera such as *Enterococcus, Pediococcus, Bacillus, Streptococcus, Lactococcus, Bacteroides, Akkermansia, Propionibacterium* and *Saccharomyces* are also considered probiotics (29, 30). The microorganisms must meet some criteria to be categorized as probiotic such as antimicrobial activity against pathogenic bacteria, resistance to gastric and bile acid, adherence to mucus or human epithelial cells, and ability to alleviate pathogen adhesion to surfaces and bile salt hydrolase activity (31). Probiotics are generally recognized as safe (GRAS) and provide diverse health benefits, including modulation of the immune system, balancing the intestinal microflora, reduction of cholesterol level and lactose intolerance, production of bioactive compounds (bacteriocins, short-chain fatty acids, B-vitamins, vitamin K_2_ and enzymes), increasing the bioavailability of nutrients, protection against pathogenic bacteria and different diseases (32–34). The main mechanisms involved in beneficial health effects of probiotics include antagonistic effects *via* generation of antimicrobial substances, competition with pathogens for nutrients and binding sites, immunomodulatory effects and prevention of toxin production by bacteria (35, 36).

The concept of prebiotics was first introduced in 1954. Gyorgy reported that N-acetyl-glucosamine, a component of human milk, caused the growth of *Bifidobacterium*. In 1957, Petuely identified lactulose as a bifidus factor. In the 1970s and 1980s, Japanese researchers reported several indigestible oligosaccharides as bifidus factor (37). Prebiotics are non-digestible carbohydrates that promote the growth of some special microorganisms in the gut (38). Some sources of prebiotics include fruits and vegetables, soybean, grains, artichoke, chicory and yacon roots (39). The most common prebiotics are fructo-oligosaccharides (FOS), galacto-oligosaccharides (GOS), and trans-galacto-oligosaccharides (40). Nowadays, polyunsaturated fatty acids and polyphenols are also considered prebiotics because they are selectively used by the host microbiome and have presented potential health benefits (41). Prebiotics help the absorption of minerals, preserve the integrity of the intestinal epithelial layer, increase resistance against pathogenic colonization and decrease the risk of large intestine cancer (42). The design of prebiotic food not only improves the probiotic viability, but also targets the production of value-added foods.

**Figure 1:**
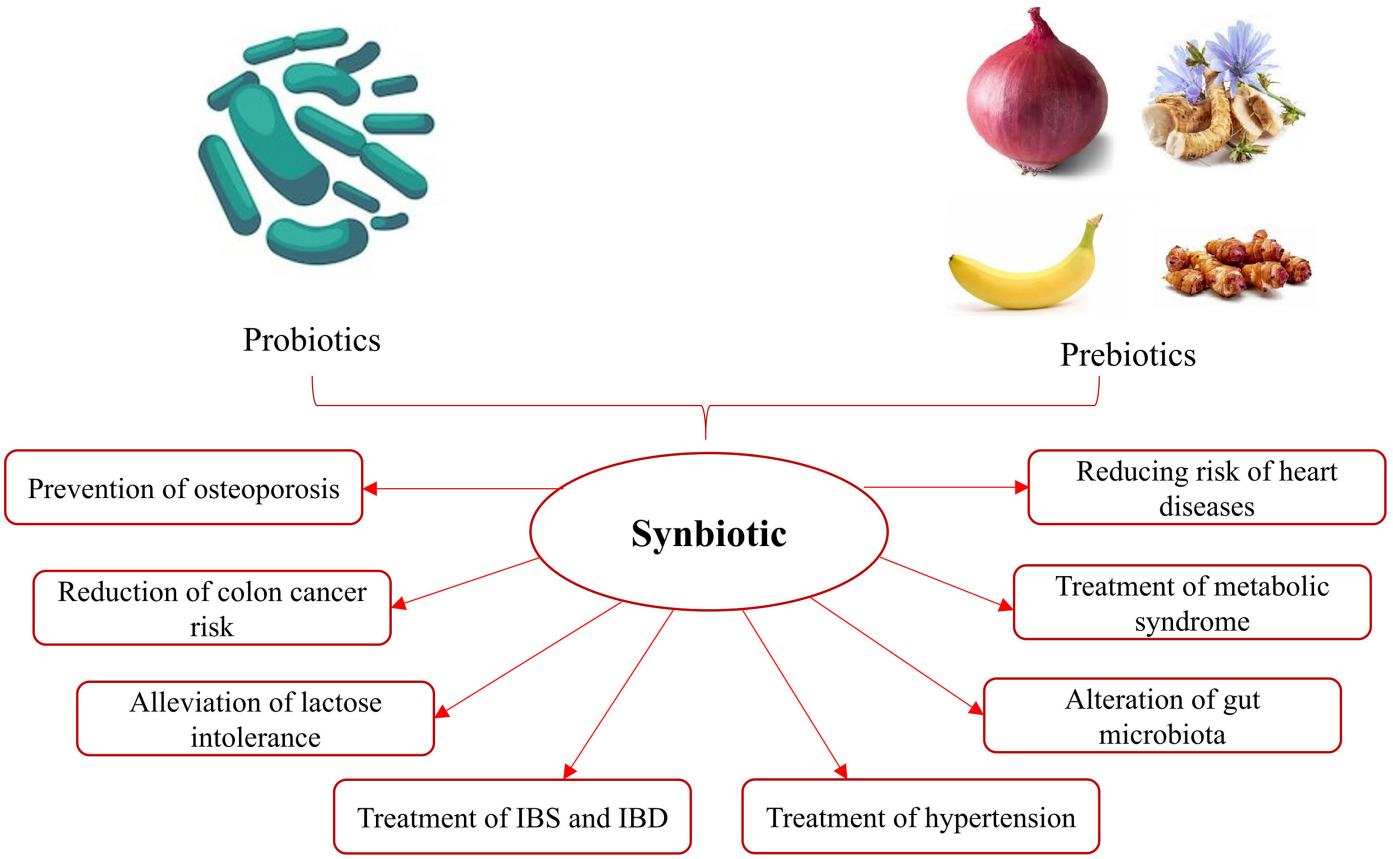
Some health advantages of synbiotic.

The word “synbiotic” describes a product consisting of probiotics and prebiotics and implies synergism. This term should be applied to products in which the prebiotic compounds selectively enhance the viability of probiotics (43). The synergistic combination of prebiotics with probiotics beneficially influences the host by improving the survival and administration of live microbial dietary supplements in the GI tract. It is pointed out that synbiotic has greater health-promoting properties than probiotics and prebiotics individually (44). Figure 1 demonstrates the health benefits of synbiotic.

## Edible films and coatings; general remarks

Along with the increased time of food consumption from farm to fork, there is a great need to package food products. Food packaging provides a hindrance against deterioration, dehydration, loss of flavor, appearance and nutritional value during handling, storage, and transportation of foods (45, 46). The most common materials used for food packaging are polymeric materials that have brought environmental concerns due to their non-biodegradability (47). Therefore, applying edible and biodegradable packaging systems (film/coating) as sustainable food packaging is a topic of considerable attraction (48). The materials used in preparation of edible packaging are renewable, recyclable, easily degradable and require minimal or no need of disposal (49, 50).

Edible packaging is described as a film or coating made of food-grade materials and is applied for enrobing different food products to improve their quality and prolong the shelf life. Although the terms “film” and “coating” are used interchangeably, they indicate different concepts. Films are usually used as a thin layer of cover or wrap, whereas coatings are directly formed on the product's surface (51, 52). Edible films and coatings should provide enough mechanical strength to keep the integrity of the products and reduce moisture loss while selectively permitting for controlled exchange of essential gases, such as carbon dioxide, oxygen, and ethylene, which are involved in respiration processes to keep the quality of products (53). Edible packaging has received several applications since it has the benefits of being consumed together with the food and do not require to be removed. Edible films and coatings can delay the deterioration of highly perishable foods and elevate their quality (54). The application of edible coatings in fruits can reduce postharvest loss, thus keeping humidity, providing brightness, controlling postharvest pathogens and decreasing respiration and transpiration rates (55). It has been reported that edible coating restricted lipid oxidation and microbial spoilage of meat, poultry, and seafood (56–59). In the case of dairy products, edible packaging control the ripening process, prevent mass transfer and improve the product's shelf life (60).

Edible films can be prepared from different sustainable materials such as proteins, lipids, polysaccharides or their combination which have received significant attention due to their non-toxic and wide availability (61–63). The ingredients for preparation of films and coatings can be extracted from animal sources (chitosan, casein, whey protein, collagen, gelatin, animal fat, etc.) or plant sources (cellulose derivatives, gum Arabic, pectin, starch, soy protein, corn protein, wax, resin, etc.) (64).

Proteins used in edible film/coating are extracted from milk (casein, whey), other animal sources (collagen, gelatin), corn (zein), wheat (gluten), soy, eggs (white egg), sorghum, pea, rice bran, cottonseed, peanut, keratin, etc. Denaturation of protein by heat, solvent and pH results in film formation. Temperatures above the glass transition (Tg) undergo the transformation of proteins by molecules disaggregation, unfolding, dissociation, and straightening; molecules are reuniting through other links while the material becomes soft, elastic, and configurable in any shape. The cooled material acquires improved properties and structure due to the new covalent, hydrogen, ionic links formed (65). It has been reported that although protein films have low water vapor permeability and tensile strength compared to other polymers, various chemical, enzymatic, and physical methods as well as combining them with water-soluble substances or some polymers have been applied to improve their functional properties (66). Polysaccharides are ubiquitous natural compounds widely used for preparation of edible films/coatings. The most common polysaccharides used for edible packaging are cellulose and its derivatives, starch, pectin, inulin, sodium alginate, chitosan, carrageenan, pullulan, gellan and xanthan (67, 68). Formation of hydrogen bonds between polymer chains creates an efficient oxygen barrier. However, hydrophilic nature of polysaccharides increases their water vapor permeability (69).

Several plant fats (sunflower oil, olive oil, corn oil, etc.), animal fats and waxes (beeswax, carnauba wax, paraffin wax, and lanolin) have been used for preparation of edible films/coatings due to their hydrophobic properties and low moisture permeability. However, it should be noted that lipids cannot form an edible film alone because of lacking repeated units in the structure and impossibility of connection through covalent bonds (70). Thus, lipids are added to film-forming solution to the emulsion-based edible film to exert more hydrophobic properties. The main drawbacks of lipid films are fragile nature, greasy texture and lipid taste which restricts their application for food packaging (66).

Protein- and polysaccharide-based films have good mechanical properties but are permeable to water due to their hydrophilic nature. In contrast, lipid-based films prevent water migration, but have poor mechanical and oxygen barrier properties. Therefore, combination of these compounds can create composite films with an improved properties (71).

**Figure 2:**
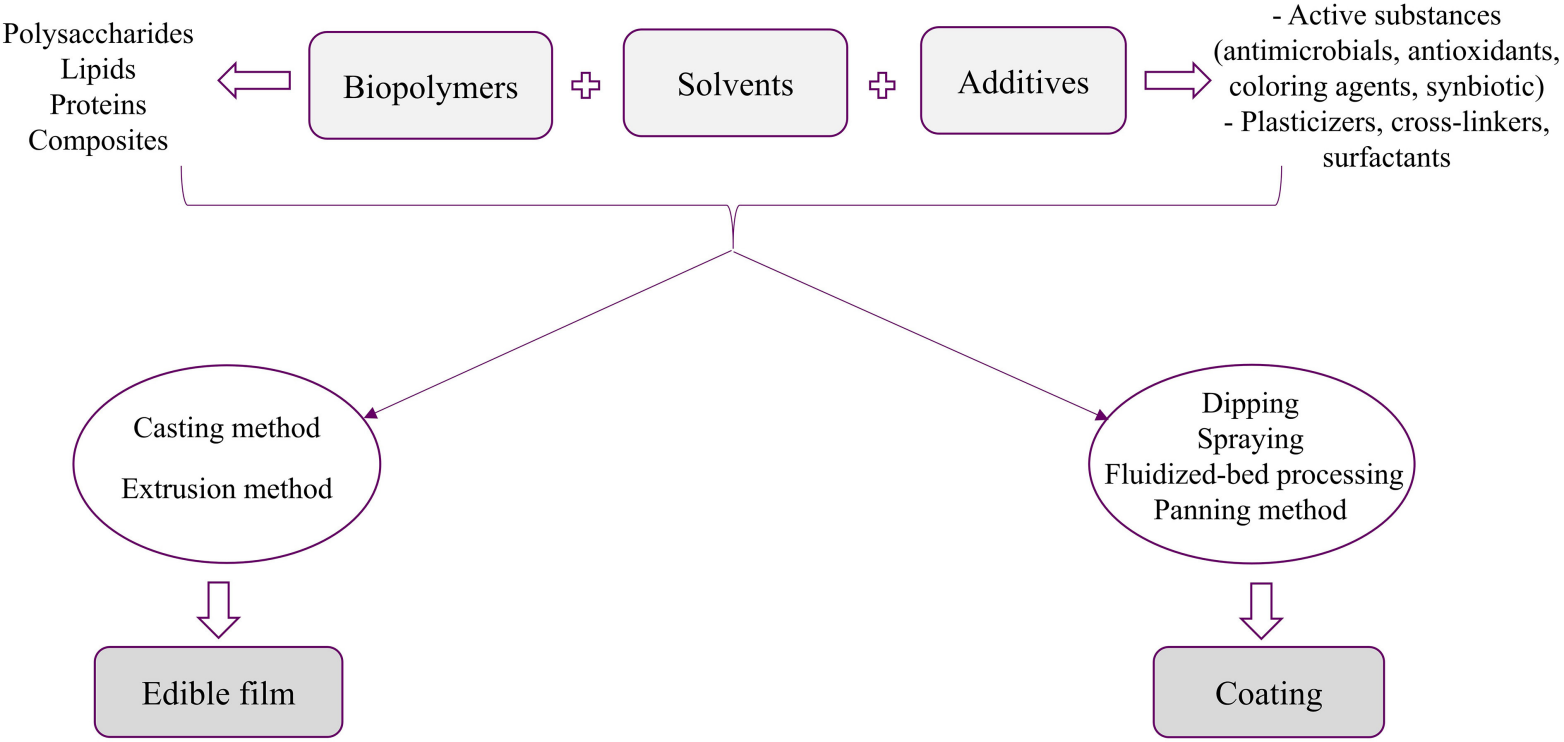
Materials and methods for preparation of edible films and coatings.

Other additives used for the preparation of edible packaging include plasticizers, emulsifiers and texture enhancers. Plasticizers are a wide range of molecules (water, polyols, fatty acids, some monosaccharides, urea, which are added to polymer material to modify the functional properties of films through increasing their extensibility, dispensability, flexibility, elasticity, rigidity and mechanical properties (72). Emulsifiers are surface-active agents which are used to facilitate dispersion of precursors and stabilize protein/lipid or polysaccharide/lipid composites and improve their adherence to food surfaces (65). Edible film/coating can also be considered as carriers for antioxidant, antimicrobial (73), nutraceuticals, coloring agents, flavors (74, 75), probiotic (76), and prebiotic (77). Hence, entrapment of these compounds in the biopolymeric matrix can increase their controlled release while reducing degradation. Figure 2 depicts the materials and methods used to develop edible films and coatings.

Two methods are used to prepare edible films, including wet and dry processes known as solvent casting and extrusion process, respectively. In the casting method, the solubility of biopolymers and additives is an essential factor, while in the extrusion technique, thermoplastic properties, phase transition, glass transition, and gelatinization should be considered. Production of edible films through the casting method involves three steps: dissolving polymers in a proper solvent (ethanol or water), spreading the solution on the mold, and drying the casted solution with an oven, microwave, or vacuum drier. This method is inexpensive without specific equipment requirements and produces more homogenous films due to the better interaction of molecules. Also, it uses low temperature that decreases the possibility of detrimental structural changes. However, long drying time, denaturation of proteins as a result of using solvents, limited forms of prepared films, production of films with different characteristics and commercialization challenges are the main disadvantages of casting method (78). In the extrusion method used at the commercial scale, a mixture of biopolymer and additive is fed to the extruder in which mixing and heating occur and an extruded film is formed (79).

Edible coatings are prepared through dipping, spraying, fluidized-bed processing and panning method. The dipping method mainly used for fruits and vegetables comprises immersion of food product into polymer solution followed by evaporation of solvent and formation of a thin layer on the product's surface (80). In the spraying method, which is the most common method, the coating solution in the form of small droplets is sprayed on the surface of the food product. The main disadvantage of this method is the impossibility of spraying polymer solutions with high viscosity (81). The panning method is putting the food product in a rotating pan and the coating solution is sprayed on the surface. The solvent is evaporated by circulated air and the dried coating is formed on the product's surface (80). The fluidized-bed method is used to form thin layers of coatings on small dry food particles and the coating solution is sprayed on the surface of products through nozzles that help to flow the smaller size food with the sprayed solution (80).

The recent studies focusing on the extension of probiotic viability have proved that using synbiotic edible packaging is a promising way to provide enough viable organisms during storage of the foods and through digestion conditions.

## Characterization of synbiotic edible films and coatings

The physical and chemical properties of edible films and coatings should be analyzed to design appropriate samples. Incorporating prebiotics and probiotics may change the characteristics of edible films and coatings. Several studies reported the insignificant effect of probiotics on the physicochemical and mechanical properties of films/coatings (82–85). However, Shahrampour et al. (86), Akman et al. (87) and Li et al. (88) demonstrated that adding probiotics significantly affected the film properties in alginate/pectin, cassava starch/CMC and alginate edible films, respectively. It was highlighted that incorporating *L. plantarum* KM 45 into alginate/pectin films reduced water vapor permeability and intramolecular space due to the formation of hydrogen bonds with film-forming substances. Similar results have been obtained by adding probiotics to cassava starch/CMC matrix. In the case of edible alginate films, incorporation of free or encapsulated *L. plantarum* increased tensile strength and water vapor permeability that were enhanced by adding encapsulated probiotics due to the hydrophilic nature of maltodextrin as the wall material. The influence of probiotics on the film properties was dependent on the composition, biopolymer structure, and intermolecular interactions (84). According to Paulo et al. (89), incorporating prebiotics into edible films and coatings resulted in plasticizing or reinforcing effects depending on the structure and level as well as increasing the viability of probiotics.

Oliveira-Alcântara et al. (90) developed synbiotic edible films based on bacterial cellulose/cashew gum containing 2% fructooligosaccharide (FOS) and *Bacillus coagulans*. It was pointed out that the addition of probiotics decreased the film strength and increased elongation explained by plasticizing effect and reducing intermolecular bonds between neighbor polymer chains resulting in increasing film flexibility and decreasing strength. Incorporation of FOS decreased both strength and elongation due to low glass transition temperature and intervening the hydrogen bonds between hydroxyl groups of the matrix. The addition of probiotic and FOS increased water vapor permeability (WVP) due to the weakening of the polymeric structure.

Zabihollahi et al. (91) prepared carboxymethyl cellulose (CMC)-based nanocomposite films containing *Lactobacillus plantarum*, cellulose nanofiber (CNF) and inulin (10 or 20%). Using cellulose nanofibers decreased the film roughness and inulin incorporation increased the compactness and density of the film's structure. The CMC film with 5% cellulose nanofiber and 20% inulin showed the highest smoothness and the lowest pores. Also, it was noted that no probiotic cells were detected on the surface of films, indicating complete coverage of cells. The highest and the lowest film thickness were obtained in CMC-based films with 2.5% CNF + 20% inulin and 5% CNF + 0% inulin, respectively. It was demonstrated that the addition of CNF or 10% inulin had no significant effect on ultimate tensile strength, but the incorporation of 20% inulin decreased tensile strength significantly. Elongation to break (ETB) in films containing 2.5% CNF and 20% inulin showed no significant difference, but the addition of 5% CNF and 20% inulin decreased the ETB values that was ascribed to the interaction of CMC with CNF or inulin and formation of a rigid network with limited mobility of polymer chains.

Sodium alginate, Arabic gum, konjac flour, pectin, and inulin have been used at a concentration of 4% to develop synbiotic edible films based on gelatin and containing Lactobacillus casei (92). The results showed that Arabic gum and inulin treatments had the least tensile strength, young modulus and moisture content, and the highest extensibility. The inulin made a distinct green tint and transparent appearance compared with the other samples.

Pereira et al. (24) prepared edible films using alginate or whey protein incorporated with *Bifidobacterium animalis* subsp. *lactis* BB-12 and prebiotics (inulin and FOS at 2%). The inclusion of prebiotics had a negligible effect on elongation at break and color properties. However, it decreased the texture resistance, water solubility, and moisture content.

Whey protein isolate (WPI) film was prepared by addition of CNF (2.5 and 5%), polydextrose (10 and 20%) and *L. plantarum* (93). It was pointed out that incorporation of CNF to WPI films improved thermal properties through the interactions with WPI, decreasing polymer mobility, interaction of CNF with water molecules and lowering its plasticizing effect and increasing film crystallinity. Cellulose nanofiber decreased the roughness in WPI films and polydextrose created a uniform and compact structure by filling the spaces within the polymer matrix. The addition of CNF and polydextrose decreased ETB and 10% polydextrose was more effective in this regard. Cellulose nanofiber and polydextrose had an increasing and decreasing effect on ultimate tensile strength due to decreased plasticity and increased elasticity, respectively.

Orozco-Parra et al. (85) incorporated *L. casei* and inulin (0, 0.1, 0.5 and 1%) into edible cassava starch film. It was indicated that elongation at break, WVP and water solubility increased by increasing the inulin level while tensile strength decreased due to the plasticizing effect and hygroscopic nature of inulin. The addition of probiotic had no significant effect on mechanical and barrier properties of cassava starch films. Regarding color parameters and opacity, no significant effect on a^*^, b^*^ and L^*^ values was observed by the inclusion of inulin and probiotic bacteria. However, the addition of probiotic bacteria increased opacity. Similarly, Pruksarojanakul et al. (25) reported an increased water solubility of konjac glucomannan films containing *L. casei* and inulin. This augmentation was explained by the hydrophilicity of inulin, the dissociation of hydroxyl groups and the formation of hydrogen bonds between konjac glucomannan and inulin. Regarding water vapor permeability, individual addition of inulin or *L. casei* had decreasing and increasing effects, respectively, while their simultaneous incorporation had no significant effect. It was pointed out that there was a direct relationship between WVP and the hydrophilic nature of inulin and glucomannan. Also, the presence of short-chain sugars in inulin structure exhibited plasticizing effect leading to a decrease in the inter-chain interactions and increasing free volume in the polymer structure. The decrease of WVP by adding probiotic was due to the hindrance of polymer chain mobility. Inulin caused a reduction in tensile strength, while probiotic bacteria had no effect.

Edible films based on duck feet gelatin containing four types of prebiotics, including Arabic gum, sago starch, dextrin and polydextrose were prepared to immobilize *L. casei* (94). The addition of prebiotics resulted in a decrease of transparency. Dextrin films showed the lowest L^*^ and the highest a^*^ and b^*^ values. The incorporation of prebiotics into gelatin films filled the spaces and pores within the matrix and created a uniform and compact structure with a complete cover of probiotics. Addition of prebiotics reduced WVP of synbiotic films except in the case of sago starch. The highest and the lowest WVP were observed in sago starch and dextrin films, respectively. The high affinity of sago starch for water, decrease of intermolecular spaces and formation of hydrogen bonds between gelatin and dextrin are the main reasons of difference in WVP values. It was also declared that proteins such as gelatin had a protective effect on probiotics by providing micronutrients and neutralizing free radicals. Also, the presence of imino acids (proline and hydroxyproline) helped to stabilize the structure of films by the formation of hydrogen bonds and immobilized the probiotic bacteria within the film. In a similar study, Soukoulis at al. (8) prepared prebiotic edible gelatin films containing wheat dextrin, polydextrose, glucose-oligosaccharides and inulin to extend the viability of *L. rhamnosus* GG. The addition of prebiotics resulted in uniform and compact films without a significant effect on film structure. Opacity increased by prebiotic incorporation and films containing wheat dextrin and inulin demonstrated the highest a^*^ and b^*^ values.

Romano et al. (10) characterized edible methylcellulose-based films containing 1, 2, 3 and 5% fructo-oligosaccharides to stabilize two strains of *L. plantarum* CIDCA 83114 and lactobacilli: *Lactobacillus delbrueckii* subsp. *bulgaricus* CIDCA 333. They found that FOS worsened the mechanical and structural quality of the films. Therefore, the concentrations of 1 and 3% were used to balance the protective effect and quality of the synbiotic films. The addition of prebiotics with a detectable increase in opacity and color values (a^*^, b^*^) was observed. Prebiotics could decrease the rigidity and texture strength and increased the flexibility of composite films. The high hydroxyl group content of prebiotics leads to an increment in the hydrophilic/hydrophobic ratio of the matrix (24, 25, 85, 95). Table 1 summarized selected publications on synbiotic edible films and coatings.

**Table 1:** Publications on synbiotic edible films and coatings and their characteristics.

**Biopolymer**	**Probiotic and prebiotic**	**Characteristics and key findings**	**Food application**	**Reference**
Gelatin	*L. rhamnosus* GG, wheat dextrin, polydextrose, glucose-oligosaccharides and inulin	Addition of prebiotics resulted in uniform and compact films with an increased opacity. 60% of probiotic cells survived during film drying at 37°C by addition of glucose-oligosaccharide. Inulin was the most effective prebiotic for protection of *L. rhamnosus* GG at 4 and 25 °C during 25 days of storage.	-	(8)
Alginate	*L. plantarum*, Lactobionic acid (20 or 40 g/L)	Synbiotic cheeses were efficient in delivering sufficient number of probiotics to the lower GIT.	Cottage cheese	(14)
Alginate	*L. rhamnosus* CECT 8361, inulin/oligofructose (80 g/kg)	Higher probiotic count was observed in blueberries with prebiotics compared to coated fruits without these fibers. Synbiotic coating had no significant effect on color and texture of coated blueberries. A reduction of 1.7 log CFU/g for *L. innocua* and no influence on *E. coli* was observed in coated fruits.	Blueberry	(20)
Gelatin	*L. rhamnosus*, inulin	Synbiotic coating increased the shelf-life of strawberries, decreased weight loss and maintained the quality of coated fruits. Moreover, the growth of yeasts, molds and aerobic mesophilic count reduced.	Strawberry	(21)
Alginate	*L. rhamnosus* and *B. animalis* subsp. *Lactis* Inulin, oligofructose (8%)	Both probiotics survived at levels above 9 log CFU/g after 8 days of refrigerated storage and maintained high viability after simulated gastrointestinal digestion. Also, antimicrobial effect of both probiotics against *L. innocua* inoculated on apple cubes was observed.	Fresh cut apples	(22)
Alginate or whey protein isolate	*B. animalis* subsp. *lactis* BB-12, Inulin (2%)	Cereal bares coated with WPI contained higher level of probiotics during storage and after *in vitro* gastrointestinal digestion as well as higher acceptability by consumers.	Cereal bar	(23)
Alginate or whey protein isolate	*B. animalis* subsp. *lactis* BB-12, Inulin and FOS (2%)	Prebiotics had a negligible effect on elongation at break and color properties. However, it decreased the resistance of texture, water solubility, and moisture content. WPI film and inulin showed better performance in maintaining probiotic viability.	-	(24)
Konjac glucomannan	*L. casei*-01, inulin (1%)	Incorporation of probiotic and prebiotic into the film caused a high water solubility, sufficient transparency, low water vapor permeability and good mechanical properties. Viability of probiotics decreased and reached below the acceptable limit after 4 days in edible films. Viability of *L. casei*-01 in coated bread buns decreased gradually with a reduction of 2 log CFU portion^−1^ after 7 days.	Bread	(25)
Cassava starch	*L. casei*, Inulin (0, 0.1, 0.5 and 1%)	It was indicated that elongation at break, WVP and water solubility increased by increasing the inulin level while tensile strength decreased. Addition of probiotic bacteria increased opacity. Inulin provided a protective environment for *L. casei*, decreasing the temperature stress and slowing the viability loss in storage and simulated gastrointestinal conditions	-	(85)
Cellulose/cashew gum	*B. coagulans*, FOS (2%)	Addition of probiotics decreased the film strength and increased elongation while FOS decreased both values and increased WVP. Higher probiotic survival was observed in synbiotic film during drying stage.	-	(90)
CMC/CNF	*L. plantarum*, Inulin (10 or 20%)	Film with 5% CNF and 20% inulin showed the highest smoothness. Incorporation of 20% inulin decreased tensile strength significantly. Addition of 5% CNF and 20% inulin decreased the ETB. Inulin increased the probiotic viability by 36% during storage. The number of pathogenic bacteria decreased in chicken filet during storage and shelf life increased.	Chicken filet	(91)
Whey protein isolate/CNF	*L. plantarum*, Polydextrose (10 or 20%)	Addition of CNF improved thermal properties of the films and decreased roughness. ETB decreased by adding CNF and polydextrose. CNF and polydextrose increased the viability of *L. plantarum* and the bacterial count increased by increasing polydextrose level. Film containing 5% CNF and 20% polydextrose showed antibacterial activity against *Salmonella enterica, P. aeruginosa, S. aureus* and *E. coli*.	-	(93)
Duck feet gelatin	*L. casei*, Arabic gum, sago starch, dextrin and polydextrose	Addition of prebiotics decreased transparency of films and resulted in a uniform structure. The highest and the lowest WVP were observed in sago starch and dextrin films, respectively. Arabic gum was the most effective prebiotic for improving the stability of *L. casei* and increased its viability by 42 and 45% at 4 and 25°C, respectively.	-	(94)

## Viability of probiotics in synbiotic edible films and coatings

One of the significant parameters of consideration for edible films is to deliver health benefits by providing viable probiotics to the gastrointestinal tract. Prebiotics has also been incorporated into edible films to improve the stability of probiotic strains. The viability of different probiotic strains under various conditions in the presence of prebiotics has been investigated in several studies.

In the development of coated strawberry with gelatin, including *L. rhamnosus* HN001 and inulin, Temiz and Ozdemir (21) reported the initial count of *L. rhamnosus* as 11 log CFU/g that reached 7.43 log CFU/g and 7 log CFU/g for samples with and without inulin, respectively, after 15 days of storage at 4°C. The results were indicative of the inulin ability to keep the probiotic viability. Viability of *L. casei* in synbiotic gelatin films was evaluated at 4 and 25°C during 25 days of storage. It was reported that survival of probiotics was lower at 25°C compared to 4°C. Arabic gum was more efficient in improving the stability of *L. casei* than other prebiotics and increased probiotic viability by 42 and 45% at 4 and 25°C, respectively (94). According to Zoghi et al. (96), the steric barrier of solutes, interaction through hydrogen bonds with polar groups of membrane phospholipids and the presence of nutrients and free radical scavenging compounds are the possible factors affecting the stability of probiotic in prebiotic films.

Sáez-Orviz et al. (14) coated cottage cheese with sodium alginate containing *L. plantarum* CECT 9567 and lactobionic acid (LBA) as prebiotic. The survival of *L. plantarum* was analyzed during 15 days of storage and in simulated gastrointestinal conditions. The coated cottage cheeses reached the minimum legal enumeration (10^6^ CFU/g cheese) to join the probiotic category. Initial microbial growth was observed in all samples (probiotic and synbiotic samples) because LBA acted as a substrate followed by a decrease and then a stabilization stage at the end of the period, possibly due to the unavailability of remaining LBA for the bacteria. The probiotic, synbiotic 2 (20 g/L LAB), and synbiotic 4 (40 g/L LAB) cheese samples reached a final concentration of 6.53, 6.72, and 7.23 log CFU g^−1^, respectively. The synbiotic samples possessed the ability to carry a sufficient number of probiotics at the lower gastrointestinal tract.

Karimi et al. (93) prepared nanocomposite films based on whey protein isolate containing cellulose nanofiber, polydextrose and *L. plantarum*. It was revealed that cellulose nanofiber and polydextrose enhanced the viability of *L. plantarum*. Different levels of cellulose nanofiber had no significant effect on probiotic viability due to the formation of hydrogen bonds between cellulose nanofiber and polar groups in membrane phospholipids ameliorated the bacterial stability. The number of probiotic cells increased by increasing polydextrose level.

Oliveira-Alcântara et al. (90) assessed the viability of spore-forming *Bacillus coagulans* in edible bacterial cellulose/cashew gum film and the presence of 2% FOS. Synbiotic film showed higher probiotic survival than probiotic film during the drying stage. Different sugar molecules explained the protective effect of FOS in its structure; the smaller sugar molecules protected lipid membrane and larger molecules created glassy state leading to restriction of molecular mobility and interactions. Storage stability was studied at temperatures of 4, 20, and 37°C for 45 days. They reported that the composite film kept a satisfactory probiotic count at all temperatures. Even at 37°C, the loss of viability was about 1 log CFU/g. The high stability of cells was probably due to the spore-forming ability, which made them much more suitable than lactic acid bacteria. The incorporation of FOS caused no significant effect on the storage stability of probiotics compared with the control sample. Therefore, the effect FOS was small enough, and the viability of *Bacillus coagulans* was already at sufficient counts.

Fresh cut apples were functionalized by prebiotic-alginate coating as a carrier for *L. rhamnosus* and *B. animalis* subsp. *lactis* (22). The effect of inulin and oligofructose at a concentration of 8% was evaluated during refrigerated storage and after simulated gastrointestinal digestion (GID). Both types of bacteria survived at concentrations above 9 log CFU/g after 8 days of refrigerated storage. The samples maintained the crucial limit of viability after simulated digestion conditions. The assay of the antagonistic effects of probiotics against *Escherichia coli* O157:H7 and *Listeria innocua* demonstrated that the coatings significantly decreased their count during storage time.

Orozco-Parra et al. (85) reported that incorporation of *L. casei* R4603008 into cassava starch film solution before the casting process had no detrimental effect on probiotic viability that was attributed to the low drying temperature (35°C) and protective effects of inulin and cassava starch. Assessing the survival of *L. casei* during 28 days of storage at 10 and 25°C showed that bacterial count decreased significantly at the first week of storage at both temperatures and two inulin levels.

During the whole storage period at 10°C, the inactivation rate reduced. After 21 days at 10°C, <75% of the initial *L. casei* survived, while at 25°C, <20% remained. It was noted that storage temperature and the presence of inulin affected probiotic viability. The survival of *L. casei* added to synbiotic film in simulated gastrointestinal conditions showed a higher reduction in simulated gastric fluid than simulated intestinal and colonic fluids, which was ascribed to the low resistance of cassava starch to low pH. However, the inclusion of inulin provided a protective environment for *L. casei*, decreasing the temperature stress and slowing the viability loss in storage and simulated gastrointestinal conditions. Similar results have been reported in CMC-based films containing *L. plantarum* ATCC 14917 and inulin (91) and gelatin-based coating containing inulin and *L. rhamnosus* (21).

Alginate-based coating supplemented with *L. rhamnosus* CECT 8361 and inulin/oligofructose was applied for fresh blueberries (20). It was observed that the probiotic count in blueberries with prebiotics (6.2 log CFU/g) was higher than in coatings without these fibers (5 log CFU/g). The antibacterial effect of coated blueberries on *E. coli* O157:H7 FP605/03 and *L. innocua* CIP 8011was also tested during storage. The obtained results indicated a reduction of 1.7 log CFU/g for *L. innocua* and no influence on *E. coli*.

Pruksarojanakul et al. (25) reported that the inulin joint in konjac glucomannan had a more pronounced effect on the viability of *L. casei*-01 than non-prebiotic film after film formation. However, at the end of 8th day at room temperature, there was a slight difference between samples. They observed a rapid loss of viability after 4 days, probably due to heat and osmotic shock during storage. Viability of *L. casei*-01 in coated bread buns decreased gradually with a reduction of 2 log CFU portion^−1^ after 7 days. The difference was attributed to the high water activity of breads and its supporting effect on survival of probiotics.

Cereal bars were coated with whey protein isolate or alginate containing *B. animalis* subsp. *lactis* BB-12 and inulin (23). Probiotic viability was examined at room temperature during 90 days of storage and after *in vitro* gastrointestinal digestion. After 40 days, the viability in alginate-coated cereal bars decreased to 8.18 log CFU/g, meaningfully lower than WPI-coated bars that remained at 8.80 log CFU/g. However, the viable cells were 8.31 log CFU/g and 7.76 log CFU/g in WPI and alginate coatings, respectively, which passed the minimum required threshold of the probiotic population (10^6^-10^8^ CFU/g) at both samples. After *in vitro* digestion in alginate-coated cereal bars, the viability was lower than the recommended range. The nature of coating materials influenced the interaction capacity between probiotic and matrices, which directly determined the range of viability.

Pereira et al. (24) incorporated inulin and FOS into whey protein isolate and alginate edible film to improve the viability of *B. animalis* subsp. *lactis* BB-12. The viability was tested during film formation and 60 days of storage at room temperature. It was reported that the drying process had no significant effect on the survival of *B. animalis* subsp. *lactis* BB-12. The viability of B. animalis subsp. *lactis* BB-12 decreased from 10^9^ CFU/g to 10^5^-10^6^ CFU/g in control films and to 10^6^-10^7^ CFU/g in prebiotic films during storage. Inulin showed higher efficiency in maintaining probiotic viability and considering film type, whey protein had better performance due to supplying more nutrient compounds, decreasing redox potential and increasing the buffering capacity.

Phovisay et al. (92) added sodium alginate, gum arabic, konjac flour, pectin, or inulin as prebiotic to gelatin to evaluate the viability of *L. casei* TISTR 1,463 at both room and refrigerated temperature during 20 days of storage. The film containing inulin kept the highest survival (87.4%) after drying, followed by sodium alginate (83.6%), konjac flour (80.3%), gum arabic (80.0%), and pectin (47.6%), respectively. Polymer structure, glass transition and pH of film solution were suggested as possible factors influencing the viability of *L. casei*. The viability was significantly higher at 4°C than at room temperature, which was derived from reduced enzymatic and chemical reactions of probiotic cells at chilling temperature. At room temperature, films with Arabic gum showed the greatest reduction rate, followed by alginate, inulin and konjac. In contrast, at 4°C, the highest reduction was observed in konjac film, followed by inulin, alginate and gum Arabic. The shelf life of synbiotic edible films was estimated to be more than 100 and 5 days at 4°C and room temperature, respectively.

Romano et al. (10) prepared methylcellulose (MC) films containing two lactobacilli strains (*L. delbrueckii* subsp. bulgaricus CIDCA 333 and *L. plantarum* CIDCA 83114) and FOS as a prebiotic. Since FOS brought adverse effects on the structural properties of the films, the FOS concentrations (0, 1, 2, 3, and 5%) were selected to equilibrate between protecting the bacteria and structural effects. The viability of films, including *L. delbrueckii* with 3% w/v FOS and *L. plantarum* with 1% FOS were tested at a relative humidity of 11, 33 and 44% at refrigerated temperature. *L. plantarum* was found to be stable for longer periods at higher RH values compared to L. delbrueckii.

Sokoulis et al. (8) developed gelatin films containing *L. rhamnosus* GG and four prebiotics. Glucose-oligosaccharide provided the best protection to the probiotic during film drying at 37°C, allowing the survival of 60% of cells, followed by 26% for polydextrose. Inulin and wheat dextrin showed a negative effect on bacterial survival. On the other hand, inulin was the most effective prebiotic for protection of *L. rhamnosus* GG at both tested temperatures (4 and 25 °C) during 25 days of storage, followed by wheat dextrin, glucose oligosaccharide and polydextrose. It has been mentioned that the temperature between the storage and glass transition temperature facilitated non-enzymatic browning and affected the viability of probiotics (97).

## Application of synbiotic edible films and coatings in food products

Only a few studies applied synbiotic edible films and coatings in food products. However, it has been reported that synbiotic packaging could maintain the microbial quality and in some cases, affected sensory properties.

In a study by Alvarez et al. (22), edible alginate coatings containing *L. rhamnosus* and *B. animalis* subsp. lactis as well as oligofructose, and inulin as prebiotics were applied to red apple cubes. Sensory analysis was performed in control (without the coating), samples with the probiotic coating, and samples with synbiotic coating. The analyzed attributes were the overall visual quality, flavor, color, and odor, scored from 0 (dislike extremely) to 5 (like extremely). At the end of the 8 days, in the synbiotic coating with *L. rhamnosus*, overall visual quality, color, and odor scored under the established acceptability limit (2.5), while scores for samples containing *B. animalis* subsp. lactis exceeded this limit indicating acceptable sensory properties. The reduction in hue parameter implied oxidative and non-oxidative reactions of polyphenols and Maillard reaction, resulting in colored condensation products and melanoidins, respectively. In another study, synbiotic-supplemented whey protein or alginate coatings were used for cereal bars (23) and it was revealed that physicochemical properties (a_w_, moisture content, color and texture) were not affected during storage. Appearance, color, odor, flavor, texture, crunchiness, and adhesiveness were analyzed using a 9-point hedonic scale by 5-point ratings, where 1 and 2 corresponded to “too weak” (TW), 3 to “just about-right” (JAR), and 4 and 5 to “too strong” (TS). The presence of *B. animalis* subsp. *lactis* BB-12 and inulin-supplemented film significantly affected the color, odor, flavor, adhesiveness, crunchiness, and cohesiveness of the product. For uncoated samples, the median of the evaluations corresponded to “liked very much.” At the same time, synbiotic whey protein and alginate-coated samples corresponded to “liked moderately.” The results showed that the odor and flavor of whey protein were more pleasant than alginate, which was due to its off-flavor.

Gelatin-based edible coating with inulin and *L. rhamnosus* HN001 was used for fresh strawberries. It was indicated that synbiotic coating reduced yeasts, molds and aerobic mesophilic bacteria counts during storage. In addition, synbiotic coating preserved strawberries' quality, total phenolic content and antioxidant activity (21).

The antimicrobial effect of CMC-based nanocomposite film containing *L. plantarum*, 2.5% CNF and 20% inulin on chicken filets was assessed. It was observed that the number of aerobic mesophilic bacteria, psychrotrophic bacteria and coliforms decreased significantly and storage time of chicken filets increased from 3 days in control samples to 6 days in samples packaged with synbiotic films (50). Production of various metabolites such as organic acids, hydrogen peroxide, ethanol, diacetyl, acetaldehyde as well as the competition of *L. plantarum* with spoilage microorganisms for nutrients were the possible antimicrobial mechanisms (98).

Utilization of alginate-based coating with *L. rhamnosus* and inulin/oligofructose in fresh blueberries had no effect on the acceptability of samples stored for 14 days at refrigeration temperature (15).

## Conclusion and future trends

Synbiotic edible films and coatings are promising bioactive packaging and a suitable carrier for probiotics and provide health benefits for consumers. The presence of prebiotic ingredients such as FOS, GOS, xylooligosaccharide (XOS), inulin and polydextrose in edible films and coatings resulted in a decrease of tensile strength, and increased elongation to break, water vapor permeability and compactness. Also, the viability of probiotics in synbiotic edible films and coatings improved during storage and simulated gastrointestinal tract. Application of synbiotic edible films and coatings in food products showed inhibitory activity against spoilage microorganisms without affecting sensory characteristics. However, further studies are required to explore other probiotic species and prebiotic compounds from different sources developing synbiotic edible films and coatings. Extraction of prebiotics from food and agricultural waste can reduce the cost of production. Utilization of resistant probiotics to harsh processing conditions and suitable encapsulation methods can increase the viability of probiotics in synbiotic edible film/coating. Another novel concept in the development of edible packaging can be inclusion of postbiotics including inanimate microorganisms or their components instead of live microorganisms. Regarding film characteristics, enzymatic treatments and fermentation along with physical processing can be investigated in order to tailor the mechanical, physical and barrier properties of edible films. Also, more research are required to optimize the method and conditions for producing synbiotic edible films and coatings on an industrial scale and assess their application in different food products such as dairy, cereal-based and meat products. Furthermore, the ability of probiotics strains to survive the gastrointestinal tract and their colonization in the intestine should be explored by *in vivo* studies.

## Author contributions

NK and MM designed the study and critically revised the manuscript. The manuscript was written by SS, NK, and MM. All authors contributed to the article and approved the submitted version.

## Conflict of interest

The authors declare that the research was conducted in the absence of any commercial or financial relationships that could be construed as a potential conflict of interest.

## Publisher's note

All claims expressed in this article are solely those of the authors and do not necessarily represent those of their affiliated organizations, or those of the publisher, the editors and the reviewers. Any product that may be evaluated in this article, or claim that may be made by its manufacturer, is not guaranteed or endorsed by the publisher.
